# Intraoral Lipoma on the Palate of an 11-Year-Old Patient: A Case Report

**DOI:** 10.3390/reports8010019

**Published:** 2025-02-08

**Authors:** Vasileios Zisis, Christina Charisi, Konstantinos Poulopoulos, Petros Papadopoulos, Athanasios Poulopoulos

**Affiliations:** Department of Oral Medicine/Pathology 1, School of Dentistry, Aristotle University of Thessaloniki, 54124 Thessaloniki, Greeceakpoul@dent.auth.gr (A.P.)

**Keywords:** oral, intraoral, palatal, lipoma, children, pediatric, lesion, tumor, benign, head and neck pathology

## Abstract

**Background and Clinical Significance**: Lipomas, benign tumors composed of adipose tissue, are recognized as one of the two most common fat-containing soft tissue tumors, underscoring their relative prevalence among benign tumors in children. Despite their prominence, lipomas rarely occur before 20 years of age, highlighting a discrepancy between their commonality and the age at which they typically manifest. This case report focuses on a 11-year-old patient who noticed the presence of an intraoral mass, which prompted further investigation, ultimately leading to the diagnosis of a lipoma located on the palate. Following our diagnosis, we searched for similar cases; however, the relevant literature was rather limited. There was a case report of a 4-year-old patient who presented with a lipoma on her tongue and a case report of a 6-year-old patient who presented with a lipoma on the buccal mucosa. **Case Presentation:** The young patient came with his parents to the Department of Oral Medicine and Pathology, School of Dentistry, Aristotle University of Thessaloniki, Greece, and reported the presence of a growth in the middle of the upper jaw. A tumor, of approximately 1 cm diameter, was observed in the middle of the palate, on the border between the hard and soft palate. The surrounding mucosa appeared normal, which is critical in differentiating the tumor from more aggressive pathological entities. It was characterized by a soft and slippery consistency. The patient was referred to a cone beam computed tomography (CBCT) examination to investigate if there was any bone involvement. Based on clinical and radiographical findings, a biopsy was carried out. The tumor was initially excised in its entirety and the base was electrocauterized to avoid placing sutures. The histopathological examination that followed suggested the presence of an intraoral lipoma since lobules of mature adipose tissue in lamina propria and fatty tissue in close proximity to mucinous salivary glands were noticed. **Conclusions:** The development of lipomas in young patients can be attributed to a multitude of factors that interplay with one another, emphasizing the need for a comprehensive understanding of these growths. Additionally, underlying conditions such as diabetes mellitus, hypercholesterolemia, and obesity also play a crucial role, highlighting the interconnected nature of metabolic disorders and lipoma formation. The surgical approaches for the removal of oral lipomas primarily revolve around complete surgical excision, which is considered the mainstay treatment for these benign tumors.

## 1. Introduction and Clinical Significance

Lipomas, benign tumors composed of adipose tissue, are recognized as one of the two most common fat-containing soft tissue tumors, underscoring their relative prevalence among benign tumors in children [1]. Despite their prominence, lipomas rarely occur before 20 years of age, highlighting a discrepancy between their commonality and the age at which they typically manifest [2]. This observation aligns with the general pattern of pediatric tumors, where benign conditions prevail, yet with a predilection for later adolescence rather than early childhood [1]. Notably, lipoblastomas, which are another form of fat-containing soft tissue tumor, account for a very small percentage of childhood tumors and primarily present in infancy and early childhood, further distinguishing their occurrence pattern from lipomas [1,2]. While lipomas and lipoblastomas are prevalent within the spectrum of fat-containing tumors, the overall incidence of such tumors is relatively low, comprising only 6% of soft tissue tumors in children, adolescents, and young adults [1]. The clinical presentation of oral lipomas in children can often be misleading, mimicking other more common oral lesions, thus complicating the diagnostic process. Treatment typically involves surgical excision, yet the approach must be carefully tailored to minimize risks and complications, particularly in younger patients who may have different anatomical considerations and healing responses compared to adults. The surgical anatomy of the soft palate shows that a fibro-fatty layer is localized in the oral part of the soft palate and becomes thick and dense along the midline [3], which in turn allows for the emergence of such lesions. This case report focuses on a 11-year-old patient who noticed the presence of an intraoral mass, which prompted further investigation, ultimately leading to the diagnosis of a lipoma located on the palate. Following our diagnosis, we searched for similar cases; however, the relevant literature was rather limited. There was a case report of a 4-year-old patient who presented with a lipoma on her tongue [4] and a case report of a 6-year-old patient who presented with a lipoma on the buccal mucosa [5].

## 2. Case Presentation

**Figure 1:**
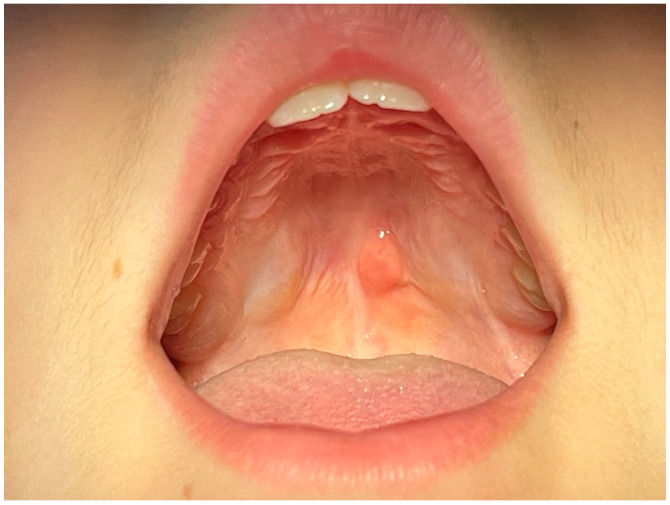
Clinical appearance of the palatal tumor.

The young patient came with his parents to the Department of Oral Medicine and Pathology, School of Dentistry, Aristotle University of Thessaloniki, Greece, and reported the presence of a growth in the middle of the upper jaw. He only experienced occasional discomfort while eating, for the past few weeks, suggesting that the symptoms were not severe enough to warrant immediate medical attention. The growth lacked any signs of pain or discharge, which are often indicators of more serious conditions. The mother of the patient signed the informed consent and the clinical examination followed. A tumor, of approximately 1 cm diameter, was observed in the middle of the palate, on the border between the hard and soft palate. The surrounding mucosa appeared normal, which is critical in differentiating the tumor from more aggressive pathological entities. It was characterized by a soft and slippery consistency. Based on these characteristics, the differential diagnosis included lipoma, sialolipoma, oral dermoid and epidermoid cysts, oral lymphoepithelial cyst, benign salivary gland tumour, mucocele, benign mesenchymal neoplasm, ranula, ectopic thyroid tissue, and lymphoma [6,7] (Figure 1).

**Figure 2:**
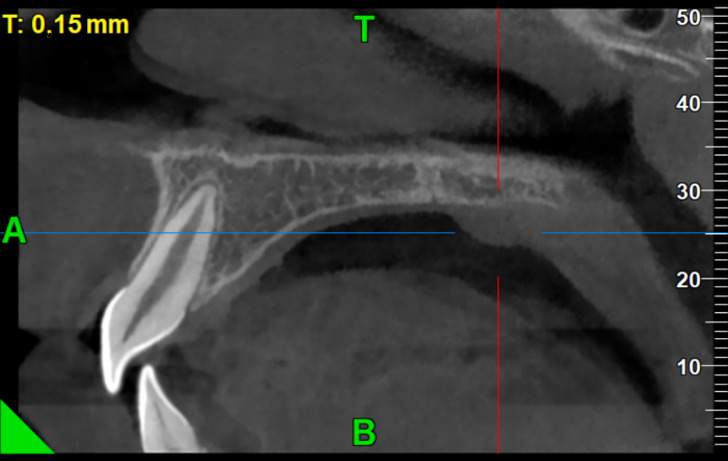
CBCT examination of the area under investigation. The lesion appears to be restricted solely in soft tissues.

The patient was referred to a cone beam computed tomography (CBCT) examination to investigate if there was any bone involvement (Figure 2).

**Figure 3:**
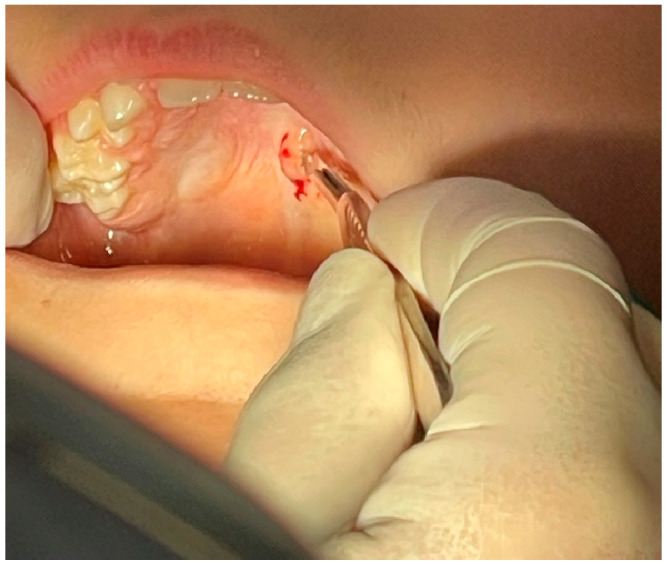
The tumor was initially excised by a scalpel.

**Figure 4:**
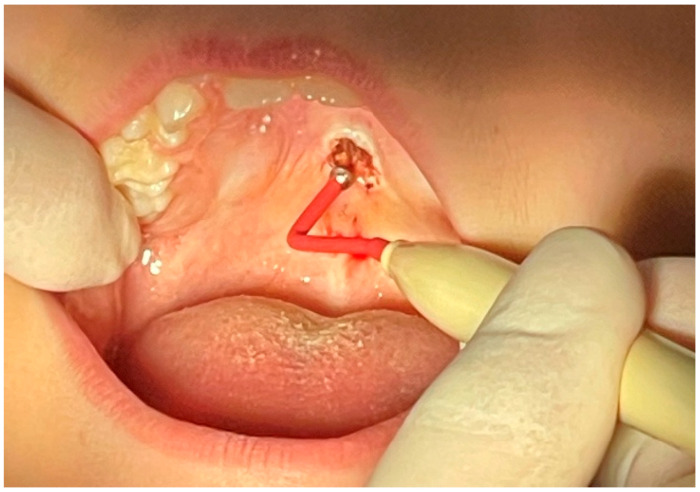
Following the excision, electrocautery was applied to prevent any postsurgical bleeding and avoid the placement of sutures.

Based on clinical and radiographical findings, a biopsy was carried out. The tumor was initially excised in its entirety and the base was electrocauterized to avoid placing sutures (Figure 3 and Figure 4).

**Figure 5:**
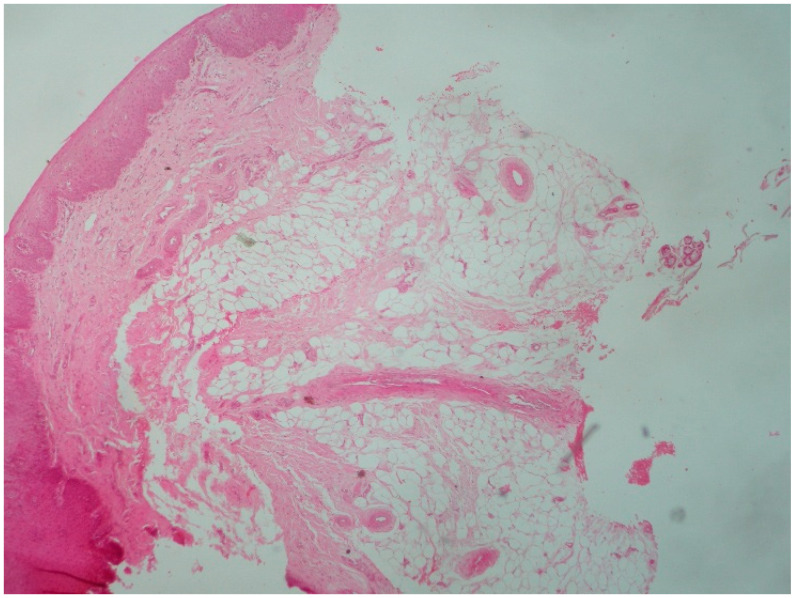
Lipoma of the palate. Lobules of mature adipose tissue in lamina propria (Hematoxylin eosin stain ×40).

**Figure 6:**
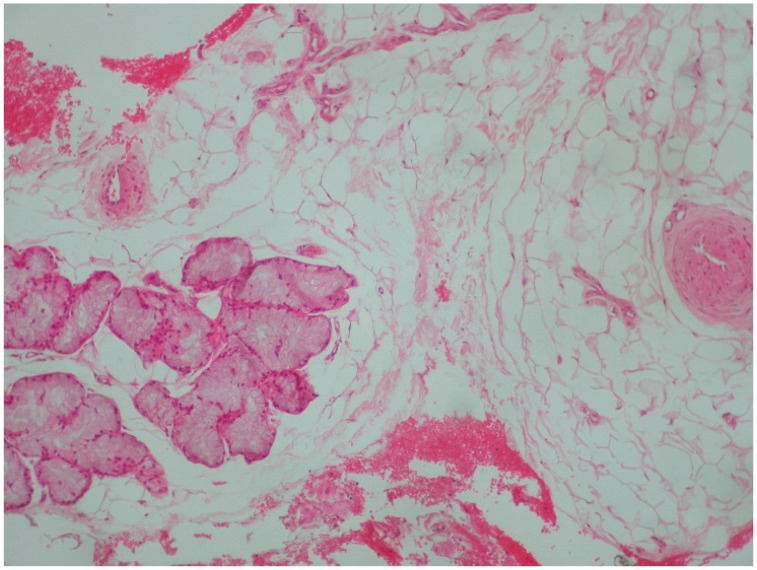
Lipoma of the palate. Fatty tissue in close proximity to mucinous salivary glands (Hematoxylin-eosin ×100).

The histopathological examination that followed suggested the presence of an intraoral lipoma since lobules of mature adipose tissue in lamina propria and fatty tissue in close proximity to mucinous salivary glands were noticed (Figure 5 and Figure 6). Sialolipoma was ruled out since a well-defined proliferation of mature adipocytes with secondary entrapment of salivary gland elements was not noticed.

**Figure 7:**
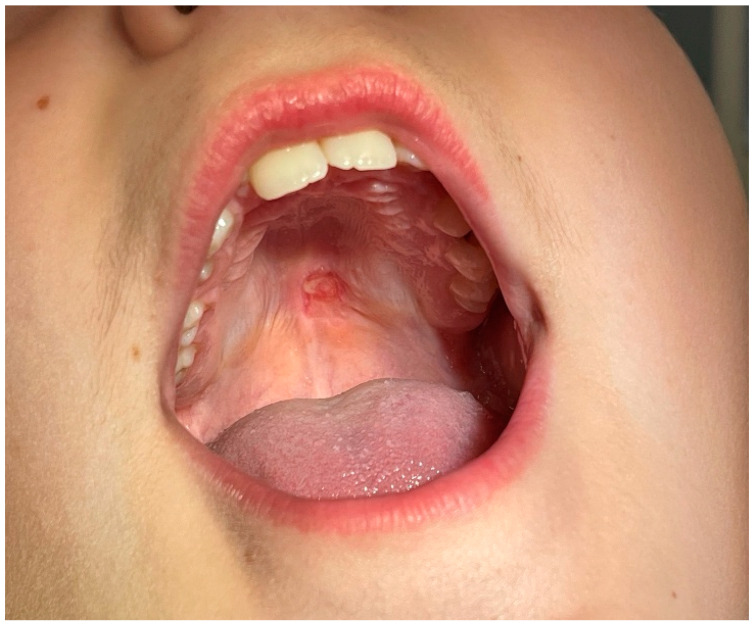
Seven days after the excisional biopsy, the healing is going as expected.

The patient was re-examined seven days later, and the healing was uneventful (Figure 7).

The patient’s parents were advised to be on the lookout for any recurrence and adhere to yearly follow ups.

## 3. Discussion

In contrast to adults, lipomatous lesions in children are relatively uncommon, accounting for only about 10% of pediatric soft tissue tumors, with lipoblastomas occurring even more infrequently [2]. The histological features of adipocytic tumors in children are distinct and display different occurrence rates compared to adults, emphasizing the need for differential diagnosis in pediatric cases to distinguish benign lipomas from more serious conditions like liposarcomas [8]. Notably, most fat-containing soft tissue tumors in children are benign, and the incidence of liposarcomas is extremely rare in this age group, which further differentiates the clinical approach needed for pediatric patients [1]. Lesions appearing as swelling on the dorsum of the tongue usually mimic hemangioma, lymphangioma, rhabdomyoma, neuroma, or neurofibroma [6].

The diagnosis of lipomas may present a challenge in pediatric cases, due to the limited lipogenic differentiation [9]. This lack of differentiation can complicate distinguishing lipomas from other soft tissue neoplasms, necessitating careful histopathological examination. Additionally, the issue is compounded when dealing with small biopsies, due to the need to minimize invasive procedures [9]. Immunohistochemistry may be of help since markers such as MDM2 and CDK4, commonly evaluated in soft tissue tumors, are typically negative in lipoma cases [9]. This absence of positive markers further complicates the differentiation process and highlights the need for a comprehensive evaluation combining clinical, radiological, and histological findings.

The differential diagnosis between lipoma and sialolipoma is based on certain features of the latter: Sialolipoma constitutes a proliferation of mature adipocytes with secondary entrapment of salivary gland elements, including serous acini, ducts, and myoepithelial cells [10]. Additionally, the differential diagnosis between lipoma and lipoblastoma is based on their histological architecture: Lipomas are composed of only mature fat without lobulation, whereas lipoblastomas show a characteristic lobular architecture, with lobules containing lipoblasts embedded in a myxoid matrix [11].

The development of lipomas in young patients can be attributed to a multitude of factors that interplay with one another, emphasizing the need for a comprehensive understanding of these growths. Additionally, underlying conditions such as diabetes mellitus, hypercholesterolemia, and obesity also play a crucial role, highlighting the interconnected nature of metabolic disorders and lipoma formation [12]. Beyond metabolic factors, familial tendencies and genetic predispositions, such as those observed in lipomatosis, underline the hereditary component in the development of these benign tumors [12]. Furthermore, trauma, particularly involving the necrosis of adipose tissue, has been implicated in triggering lipoma growth, indicating that physical factors, alongside genetic and metabolic ones, contribute to their formation. Addressing these various elements through targeted interventions and lifestyle modifications may provide a pathway to reduce the incidence and progression of lipomas in this demographic.

The surgical approaches for the removal of oral lipomas primarily revolve around complete surgical excision, which is considered the mainstay treatment for these benign tumors [13,14]. Pediatric cases present a lower complication and local recurrence ratio compared to adult cases [15]. The choice of surgical method often depends on the size and extent of the lipoma, as well as the preferences of the patient, with procedures being performed either in an office setting under local anesthesia or in an operating room for more extensive cases. Moreover, in cases where surgical intervention may not be immediately necessary or desired, injectable steroids, specifically a 1:1 mixture of lidocaine and triamcinolone acetonide, can be administered monthly to manage the size of the lipoma, potentially reducing the need for immediate surgery [13]. Ultimately, the successful and complete excision of the lipoma is essential, as it ensures there is no recurrence of the tumor, offering patients a definitive resolution to the condition [13]. In cases similar to ours, caution is required to avoid damaging the surrounding minor salivary glands and the underlying vasculature.

CT or a CBCT is usually not required for oral lipomas due to their typical presentation and minimal risk of infiltration, although it may be considered in cases where the lesion appears larger or there is a need to evaluate possible involvement of deeper tissues, as in our case [16]. On one hand, accurate identification is necessary, whereas on the other hand, exposure to unnecessary procedures should be avoided. The unique tissue composition in younger individuals may necessitate radiological evaluations, such as when assessing the proportion of fat tissue in comparison to myxoid tissue [17]. Unlike older children, infants predominantly exhibit myxoid tissue with minimal adipose content, necessitating a treatment plan that accounts for these specific tissue characteristics [17]. This differentiation is vital as the presence of myxoid tissue could influence surgical decisions, prompting a more proactive operative approach rather than a conservative ‘wait and see’ attitude to avoid potential disfigurement [17].

A critical area of investigation is the identification of specific genetic alterations that contribute to the development of pediatric lipomas, such as those involving the loci 12q14–15 and 6p21, which are known to lead to the overexpression of genes like HMGA2 and HMGA1 [18]. These loci alterations are pivotal as they provide insight into the molecular mechanisms that drive adipocytic neoplasms, potentially leading to targeted diagnostic and therapeutic strategies that could be more effective than current approaches, especially in treating cases with recurrent or multiple lipomas [18]. Moreover, understanding the molecular patterns and associated syndromes can significantly enhance therapeutic and prognostic capabilities, allowing clinicians to better predict tumor behavior and treatment outcomes in pediatric patients [18]. Research focusing on the overexpression of PLAG1 due to 8q11 locus alterations in lipoblastomas could also reveal crucial information about the genetic causes of these tumors, informing the development of personalized treatment regimens [18].

The limitations of our study include the lack of genetic and metabolic investigations in our case because they are not available on site in our healthcare facility.

## 4. Conclusions

Given these dynamics, it is essential to maintain awareness of the benign nature of lipomas and similar oral conditions while also considering the rare instances of malignant transformations, ensuring that appropriate monitoring and management strategies are adopted for early detection and intervention where necessary. Addressing these diagnostic challenges requires a multidisciplinary approach that may involve pediatric specialists, radiologists, and pathologists working in tandem to ensure accurate diagnosis and effective management of lipomas in pediatric cases.

## Data Availability

The original data presented in the study are included in the article, further inquiries can be directed to the corresponding author.
